# Investigation of the palatal soft tissue volume: a 3D virtual analysis for digital workflows and presurgical planning

**DOI:** 10.1186/s12903-022-02391-z

**Published:** 2022-08-23

**Authors:** Anna Seidel, Christian Schmitt, Ragai Edward Matta, Mayte Buchbender, Manfred Wichmann, Lara Berger

**Affiliations:** 1 Department of Prosthodontics, University Hospital Erlangen of Friedrich-Alexander University Erlangen-Nürnberg (FAU), Glueckstrasse 11, 91054 Erlangen, Germany; 2 Department of Oral and Maxillofacial Surgery, University Hospital Erlangen of Friedrich-Alexander University Erlangen-Nürnberg (FAU), Glueckstrasse 11, 91054 Erlangen, Germany

**Keywords:** Hard palate, Maxilla, Reference values, Cone-beam computed tomography, Oral surgical procedures, Connective tissue, Imaging, Three-dimensional, Soft palate

## Abstract

**Background:**

In mucogingival and implant surgery, an autologous soft tissue graft from the palate is the gold standard for reconstructing missing keratinised soft tissue and volume. Previously, presurgical measurements of the graft harvesting site were described with two-dimensional (2D) linear measurements. The present observational clinical study aimed to evaluate a three-dimensional (3D) measurement method for determining the present palatal soft tissue volume for each patient individually.

**Methods:**

Pre-existing cone beam computed tomography (CBCT) scans of 20 patients were converted into 3D Standard Tessellation Language models of the bone surface. Intraoral impressions of the maxilla were taken and digitised to visualise the gingival surface. The resulting virtual models of bone (reference value) and gingival (actual value) surfaces were merged, with tooth surfaces used for registration. The region between the central incisors and the hard palate was subdivided into 5 regions of interest (ROIs). The distance between palatal bone and gingival surface was analysed both volumetrically and linearly, and the results were statistically evaluated for the ROIs.

**Results:**

The average gingival surface area on the palate was 19.1 cm^2^, and the mean volume was 58.2 cm^3^ (± 16.89). Among the ROIs, the mean linear value was highest in the most distal region, from the second molar to the hard palate (4.0 ± 1.09 mm) and lowest in the canine region (1.9 ± 0.63 mm). For mean distance, significant differences were found for the anterior palate and the most posterior palate in comparison with all other ROIs (*p* < 0.01). The volume measurements also declined significantly and steadily between the posterior (1.9 ± 1.0 cm^3^) and anterior palates (0.4 ± 0.2 cm^3^).

**Conclusions:**

By merging digital data, palatal soft tissue could be quantified virtually. The results were reliable and comparable to previous findings with linear measurement methods. This 3D soft tissue volume analysis method fully exploited the diagnostic potential of data that are frequently collected for presurgical planning in oral surgery (i.e., CBCT + surface scans). This evaluation method might be useful for volumetric and linear measurements in other applications in anatomy and for determining palatal soft tissue dimensions in the planning stage before surgical interventions.

***Trial registration*:**

This observational clinical trial was retrospectively registered in the German Clinical Trials Register, reference number: DRKS00023918.

## Background

A healthy gingival architecture, with a harmonic red-white-margin on teeth and implants, is strongly associated with aesthetics for both the layperson and professionals [1]. Healthy gingival architecture can be impaired by gingival recessions. Periodontal and peri-implant recessions represent a multifactorial condition [2, 3], which can progress, regardless of the patients’ oral hygiene [3–5]. Previous studies have shown that more than 50% of a sample of the adult population experienced mid-buccal recessions [3, 6]. Recent findings have shown that the prevalence of gingival recessions was over 90% in the US population (30 years and older) [7]. In addition to effects on the aesthetics, attachment loss and insufficient integrity of the periodontal soft tissue complex can entail both non-carious and carious lesions in the roots and dentinal hypersensitivity [8, 9]. Furthermore, a narrow keratinised gingival band may limit efficient oral hygiene, and thus, impede the prevention of periodontal and peri-implant disease. To restore healthy, aesthetic conditions, mucogingival surgery may be considered to widen and thicken the keratinised tissue [10, 11].

Soft tissue grafting is an essential part of plastic periodontal and implant surgery. Despite all efforts to find alternative augmentation materials for mucogingival replacement grafts, autologous tissue remains the gold standard for augmenting keratinised tissue, reducing recession, and reconstructing the papilla [12, 13]. A critical aspect of surgical success is the quantity and quality of the obtained transplant tissue, in addition to many other factors, like blood supply and proper flap management [14]*.* The maxillary tuberosity and lateral palate are the most commonly used donor sites for both free gingival grafts and subepithelial connective tissue grafts [15]. The area between the first premolar and second molars is considered the most suitable harvesting site [16], due to the high ratio of tissue thickness to keratinised gingiva. However, gingival thickness and histologic composition can vary among individuals, and even within the same patient [17, 18].

The success and long-term results of soft tissue grafting can be improved by careful presurgical planning. The amount of retrievable tissue should be determined and potential adaptations of the surgery technique should be considered [12, 14]. The amount of retrieved tissue directly influences the treatment outcome, because the soft tissue graft shrinks during remodelling in the healing process. Therefore, the transplant needs to exceed the desired volume by about 30–40% [19, 20]. When covering multiple recessions, or when particularly large volume deficits are to be compensated with soft tissue, a large amount of soft tissue from the palate may be required for grafting [21]. The obligatory clinical measurement of tissue thickness at the transplant donor site before surgery can be performed either directly or indirectly [22]. A direct, but invasive measurement is bone sounding, with a needle or periodontal probe. This measurement is typically performed immediately before the procedure to avoid unnecessary anaesthesia [15]. Alternatively, indirect, atraumatic measurements can be performed by evaluating sectional images of three-dimensional radiographs [23–27] or ultrasound scans [18, 28]. Ultrasound evaluations are valid, both for determining mucosa thickness and for detecting palatal vessels. The currently available technique is to perform two-dimensional (2D), punctual linear distance measurements to estimate thickness from the bone surface to the gingival surface.

Diagnostic and therapy planning with three-dimensional (3D) digital solutions have come an integral part of modern dentistry, particularly in the fields of implant and periodontal surgery, maxillofacial surgery, and prosthodontics. Virtual assessment and preoperative planning possibilities provide the surgeon with rich information about the clinical situation and a better spatial picture of the anatomy. The modality mainly used for 3D radiological imaging in dental applications is cone-beam computed tomography (CBCT). From CBCT and Multi-Slice Computed Tomography (MSCT) data, virtual models of the patient’s clinical situation can be created, and hard tissue can be displayed in a reliable, geometrically correct way [29]. When a digital workflow is employed to carry out the patient's oral rehabilitation, the preoperatively acquired data typically consist of a CBCT scan and an intraoral surface scan, or digitised model cast, to obtain a precise display of the teeth and soft tissues. These Standard Tessellation Language (STL) models can be merged by matching their respective surfaces (e.g., the teeth). This technique is frequently used to analyse the available osseous tissue for implant placement in the desired prosthetic position and can help to make the treatment more accurate and more predictable [30]. A variety of commercial planning software is available that guides the clinician through the correct model merging process and allows virtual planning for guided implant placement [31]. Anatomical variations can be taken into account in advance, during therapy planning, and potential precautions can be identified to ensure an optimal result with minimal risk to the patient.

### Objectives

Although the benefits of preoperative procedure planning are evident, less attention has been paid to the use of 3D planning for soft tissue applications in mucogingival surgery. Therefore, the present study aimed to test the possibility of using a 3D evaluation technique for providing information about maxillary soft tissue. We used data commonly collected for navigated oral surgery and digital workflows (CBCT bone model + intraoral surface model) to perform a virtual analysis of the palatal masticatory mucosa. We hypothesised that the results would enrich the preoperative planning stage with an additional step: the investigation of the patient’s anatomical condition, with regard to the palatal soft tissue volume.

## Methods

### Study design and inclusion criteria

**Fig. 1:**
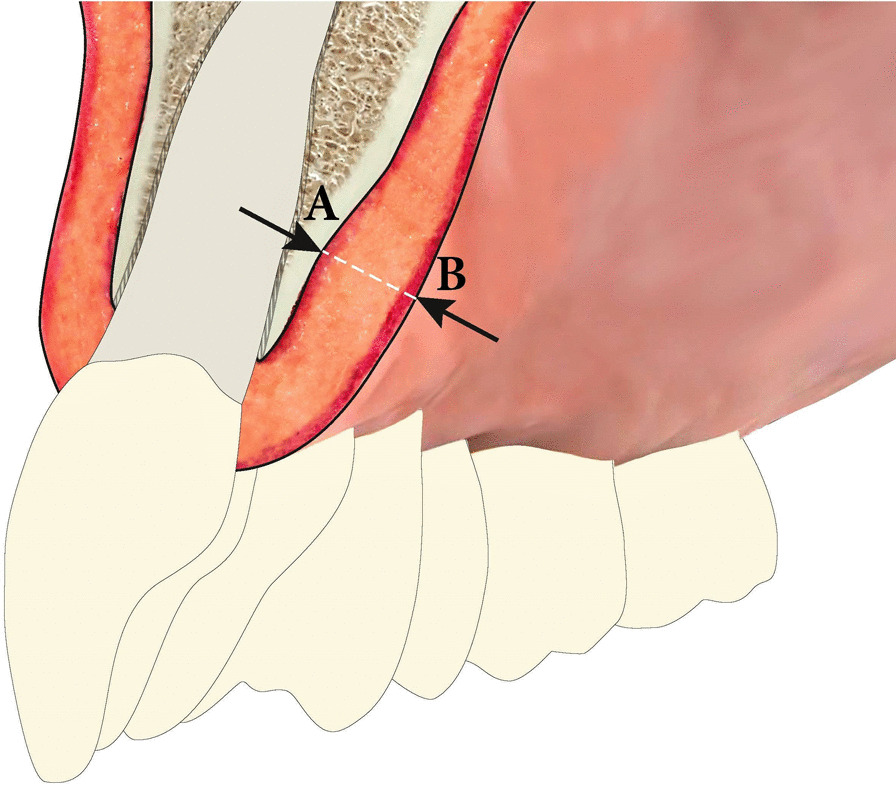
Schematic illustration of the palatal soft tissue measured in the anterior maxilla. The white dashed line indicates the space measured between **A** the bone surface and **B** the gingival surface

This prospective observational study aimed to determine individual volumes of palatal mucosa and the variation in the palatal soft tissues among patients. The study design was to merge STL models, derived from CBCT Digital Imaging and Communications in Medicine (DICOM) data, with virtual intraoral models (Fig. 1). We measured the surface discrepancies and examined correlations with findings from previous studies that measured palatal soft tissue thicknesses.

Based on the processing of digital data sets, this clinical investigation aimed to obtain as much information as possible for the presurgical planning stage by analysing the patient’s existing 3D data (e.g., from tests indicated and performed for navigated implant placement).

Patients were identified with consecutive sampling in the Department of Prosthodontics of the University Hospital Erlangen of Friedrich-Alexander University, Erlangen-Nürnberg, from November 2018 until February 2019. Eligible subjects were healthy, aged 18 -75 years. Inclusion criteria were: a complete fixed natural dentition of at least 14 teeth in the upper jaw; healthy periodontium (pocket probing depth < 4 mm) with no previous mucogingival and/or osseous surgery in the region of interest; no medication that might cause gingival hyperplasia; and a pre-existing CBCT scan (0.3 voxel size) of the entire upper jaw and palate, acquired within the prior 12 months. To avoid scattering in the CBCT images, and hence, more accurate data for merging [32], patients were excluded when metallic or ceramic restorations were found in the upper jaw. Finally, 20 patients fulfilled the inclusion/exclusion criteria and provided informed consent for participation in the study. This study was conducted in accordance with the Declaration of Helsinki on medical protocol and ethics [33], and it was approved by the Medical Ethics Committee of Friedrich-Alexander University Erlangen-Nürnberg (Approval-Nr. 350_18B). It was retrospectively registered in the German Clinical Trials Register with the reference number DRKS00023918.

### Digital data acquisition

**Fig. 2:**
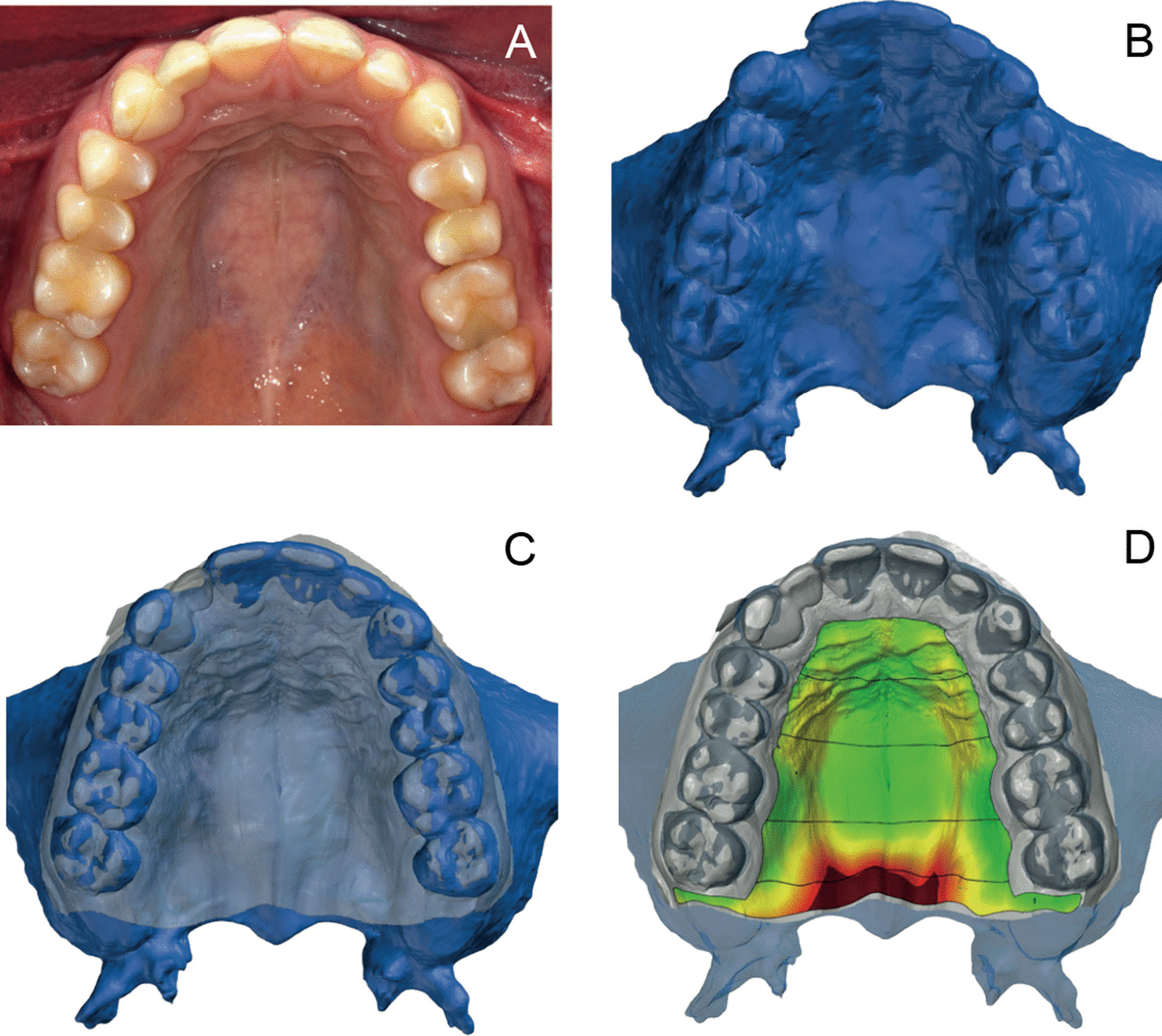
Virtual models merged to evaluate the volume of palatal soft tissues. **A** Clinical situation; **B** CBCT bone model; **C** merged CBCT and intraoral surface model; **D** colour-coded distance map representing the deviation between the two model surfaces at defined regions of interest (areas between the horizontal lines). Colour coding ranges from green (contacting model surfaces) to dark red (model surfaces separated by a large distance)

All 20 patients had undergone 3D imaging for treatment at the Department of Oral and Maxillofacial Surgery and/or the Department of Prosthodontics, University Hospital, Erlangen. Thus, all CBCTs were performed for therapeutic reasons. The CBCT scans were acquired with the following parameters: 0.3 mm voxels, 120 kV, 18.54 mA, and 8.9 s (acquisition time) (3D eXam, Kavo Dental GmbH, Biberach, Germany). The resulting DICOM datasets were converted into STL files, with Hounsfield Units (HU) for bone (threshold 150–2000 HU). The STL files represented virtual 3D models of the bone surface (Impact View 4.4.1, CT Imaging GmbH, Erlangen, Germany). The scans were precisely segmented, and small remaining holes were manually closed. The result was a relief model of the maxillary bone, with its alveolar processes and teeth, the complete vestibular site, and the palate, up to the spina nasalis posterior (Fig. 2b).

All study subjects were clinically examined. Then, a high-precision impression was taken of the maxillary region to obtain a precise model of the gingival surface of each patient’s palate for this investigation (Fig. 2a). To avoid plaster model fabrication and potential associated errors, a vinylsiloxanether (Identium® Scan Medium, Kettenbach, Eschenburg, Germany) was chosen for the impression material. The impressions were digitised directly with an industrial, high precision, optical scanner (Atos SO II, GOM GmbH, Braunschweig, Germany), with an applied measuring volume of 38 × 29 × 15 mm. To obtain the maxillary relief, the derived digital model was inverted with computer-aided design (CAD) analysis software (GOM Inspect, GOM GmbH, Braunschweig, Germany).

A CBCT bone model (in STL file format) and a virtual impression model (in CAD file format) of the upper jaw were prepared for each patient. These models were subsequently uploaded into CAD analysis software (GOM Inspect) for evaluation.

### Digital data evaluation

The maxillary CBCT model and the intraoral surface model were evaluated for each patient (Fig. 1). After importing the CBCT data into the investigation software (GOM Inspect), the CBCT model (bone) was chosen as the reference value. Then, the corresponding impression model (teeth and gingival surface) was imported into the software and designated the actual value. Next, the two models were superimposed, with the teeth as the reference surface for registration (Fig. 2c). The *matching workflow* procedure was as follows: after a rough manual alignment, the software merged the corresponding bone and gingiva models precisely, based on the registration algorithm ‘local best-fit’ over the selected tooth surfaces [34].

The distances between the virtual bone and virtual gingival surfaces were then measured (Fig. 1). This 3D-analysis enabled visualisation of the distances between the mucosal surfaces and the bone surfaces, which was equivalent to an analysis of the quantity of soft tissue (Fig. 2d). To display and measure the volume of masticatory soft tissue, a segmentation method was applied, and in each segment, a 3D surface comparison was performed between corresponding reference and actual values.

The overall region of interest (ROI) was defined as the entire area from the anterior to the posterior palate. A paramarginal distance was set to 3 mm from the teeth’s palatal sulcus. A posterior border was chosen as the line behind the second molars, which followed the margin from the hard to soft palate. The ROI was then subdivided into 5 equally sized ROIs (Table 1, Fig. 2d), which were defined in advance to facilitate conclusions about the different palatal sections.

**Table 1:** Definition of the subdivided regions of interest (ROIs 1–5)

*ROI 1*	palatal area from the anterior teeth to the mesial aspect of the first premolar
*ROI 2*	mesial aspect of the first premolar to the palatal cusp of the second premolar
*ROI 3*	palatal cusp of the second premolar to the distal fissure of the first molar
*ROI 4*	distal fissure of the first molar to the distal fissure of the second molar
*ROI 5*	distal fissure of the second molar to the posterior boundary of the hard palate

The surface discrepancy between the bone model and the gingival relief model was calculated. To visualise local volumetric differences, the discrepancies were displayed upon the rendered volume as a colour-coded distance map (Fig. 2d).

### Statistical analysis

Statistical data analyses were performed with the statistical software, R V3.6.3 [35]. For all 20 patients, the overall deviation between the bone and soft tissue surfaces was assessed with the merged STL data. The descriptive characteristics of ROIs 1–5 are expressed as the mean distance (MeanDist), minimal distance (MinDist), maximal distance (MaxDist), and volume (Vol). The Wilcoxon signed-rank test was performed to compare the MeanDist and Vol values among the subdivided ROIs. P-values were corrected with the Benjamini–Hochberg method for multiple testing. A corrected p-value < 0.05 was considered significant.

## Results

The patients had a mean age of 36.5 ± 13.1 years; the mean ages for women and men were 31.75 ± 6.4 years and 37.75 ± 14.2 years, respectively. The distances between the bone and gingival surfaces over the entire palatal region (ROIs 1–5) was examined for all 20 test subjects. Descriptive statistics are summarised in Table 2.

**Table 2:** Calculated distances between bone and gingival surfaces of the palate for the five areas of interest (ROIs 1–5)

Measurement	ROI 1	ROI 2	ROI 3	ROI 4	ROI 5	total
Area of valid distance [mm^2^]	193.4	382.9	488.5	411.9	439.5	1911.2
Mean distance [mm]	1.9	2.4	2.7	2.9	4.0	2.8
± SD	0.63	0.57	0.65	0.86	1.09	0.61
Minimal distance [mm]	0.5	0.4	0.4	0.4	0.3	0.1
± SD	0.59	0.48	0.38	0.41	0.40	0.18
Maximal distance [mm]	3.8	5.0	5.6	7.0	8.7	8.6
± SD	0.58	1.20	1.40	1.64	1.86	1.52
Volume (Vol) [mm^3^]	351.2	977.1	1392.8	1243.8	1862.3	5824.9
± SD	204.66	313.98	463.12	384.10	965.05	1689.36
Ratio: Volume per surface area [mm]	1.83	2.55	2.85	3.02	4.23	3.04

*SD* standard deviation

**Fig. 3:**
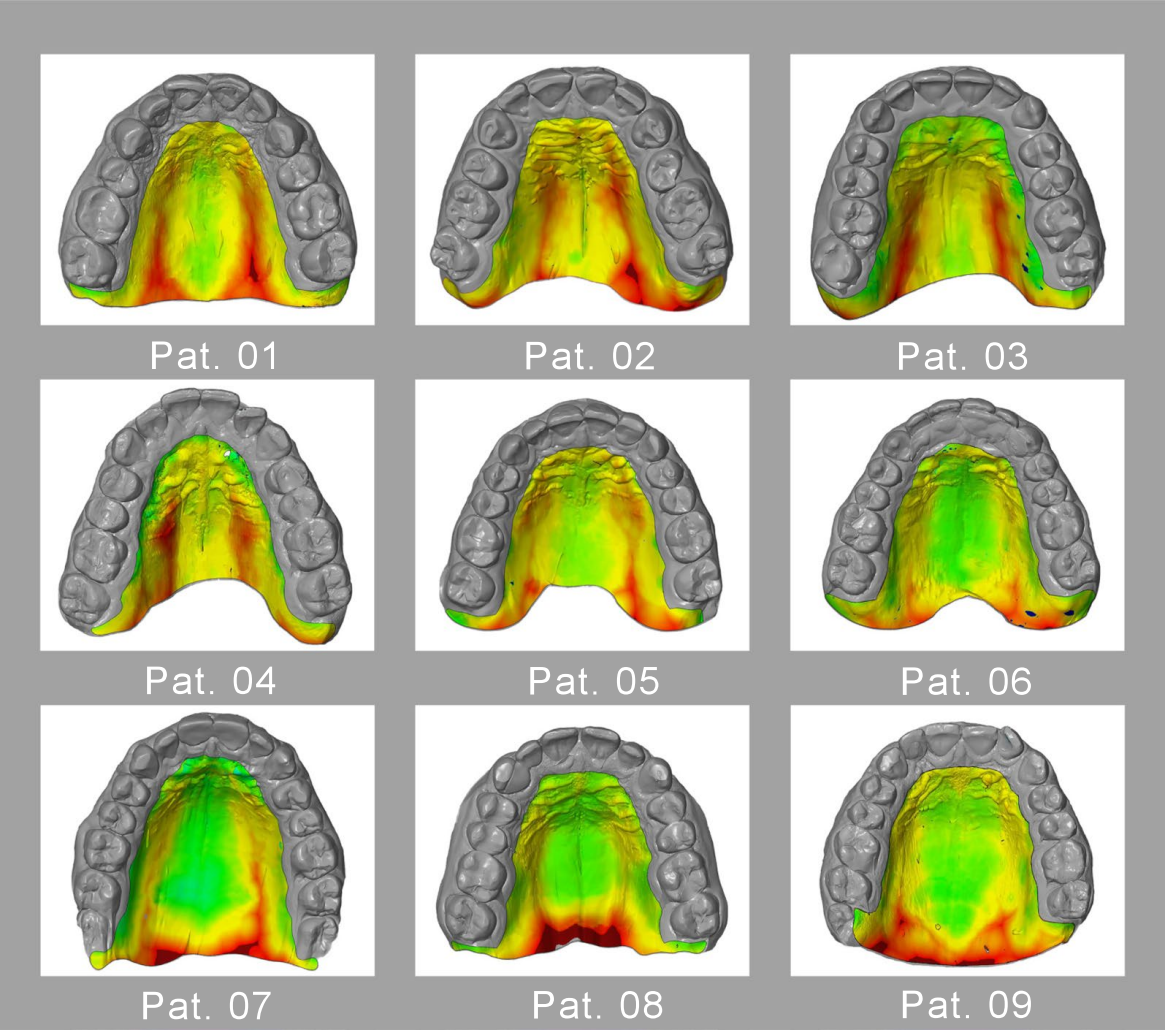
Colour-coded distance maps represent the distribution of palatal soft tissue volumes. Distance measurements are for 9 individuals. Colour coding ranges from green (contacting model surfaces) to dark red (model surfaces separated by a large distance)

In assessing all 20 individual measurements, the mean linear distance (MeanDist) of palatal bone to gingival surface was 2.8 mm (± 0.61 mm) over the entire palate. The mean MaxDist was 8.6 mm and the mean MinDist was 0.1 mm. The distribution of distances in the sample is demonstrated in Fig. 3, which shows examples of colour-coded distance maps.

**Fig. 4:**
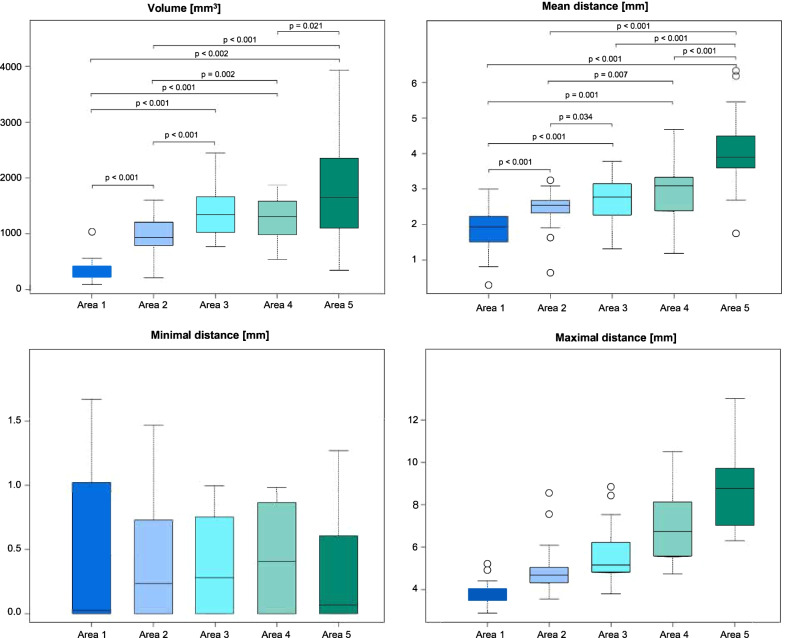
Three-dimensional (volumes) and two-dimensional (distances) measurements of maxillar palatal regions. Boxplots show the mean, minimal, and maximal distances [mm] and volumes [mm^3^]. Calculated p-values are given above each bracket; brackets indicate the two groups compared

The distances measured in individual sections of the palate (ROIs 1–5), subdivided from anterior to posterior, are shown in Table 1 and Fig. 2c. The MeanDist increased from segment 1 to segment 5, as follows: 1.9 mm in ROI 1; 2.4 mm in ROI 2; 2.7 mm in ROI 3; 2.9 mm in ROI 4; 4.0 mm in ROI 5 (Fig. 4).

The average surface area of the 20 analysed palates was 1911.2 mm^2^, with an average volume of 5824.9 mm^3^. The volume measurements in the most posterior part of the palate, ROI 5 (1862.3 mm^3^), showed the greatest degree of variation **(**SD ± 965.05). The ratio of palatal tissue volume to the palatal surface area in each ROI increased from the anterior (ROI 1, 1.83 mm) to the posterior (ROI 5, 4.23 mm) region.

The Vol and MeanDist measurements were compared between the subdivided regions (ROIs 1–5; Fig. 4). In the Vol analysis, all ROIs were significantly different, except ROIs 3 and 4 (p = 0.054) and ROIs 3 and 5 (p = 0.056). In the MeanDist analysis, only ROIs 3 and 4 were not significantly different (p = 0.143).

## Discussion

Periodontal plastic and implant surgeries are complex, technique-sensitive procedures that require a high level of expertise [36]. Hence, various factors should be addressed in the preoperative stage to ensure optimal outcomes [37]. For a soft tissue graft, the planning phase of a mucogingival surgery procedure should consider the tissue quantity and the optimal donor site [14]. The present study aimed to use the data typically collected in backward planning or navigated oral surgery (CBCTs and surface scans) for performing a volumetric digital soft tissue analysis. The currently established measurement methods for determining intraoral tissue thickness include direct and indirect procedures. The "classic" method is a direct, invasive bone sounding, with a needle or periodontal probe [15, 38]. However, the data obtained are limited, because only a few punctual linear measurements are made, and those may be biased by the uneven structure of the alveolar bone or a non-perpendicular position [26]. Due to the necessary anaesthesia, bone sounding is typically performed intraoperatively; consequently, preoperative planning based on tissue thickness is not applicable. Later studies pointed out the need for an indirect technique, with radiological imaging or ultrasound examination, which could be performed preoperatively [23, 24, 27, 28, 39–41].

The present clinical study developed a method for obtaining a volumetric, 3D representation of the palatal soft tissues. Moreover, with this method, patients were spared unnecessary anaesthesia. In the present study, no direct measurement was performed as a control that could be compared to the obtained data, because only two-dimensional data (linear measurements) were available for comparisons. Indeed, to date, no other 3D evaluation of palatal soft tissue volume has been described in the literature. Therefore, to put our MeanDist results (Table 2) into the context of the linear measurements found in the literature, appropriate articles were selected manually (Table 3) to provide an overview of the typical 2D measurements performed and mean results for the palatal mucosa.

**Table 3:** Overview of results from studies that analysed palatal soft tissue thickness

Author	Title	Year	Measurement method and site	Mean palatal masticatory soft tissue thickness [mm] ± SD for the overall palate, the first premolar region (P1) and the second molar region (M2)
Studer et al. [15]	*The thickness of masticatory mucosa in the human hard palate and tuberosity as potential donor sites for ridge augmentation procedures*	1997	Direct bone sounding with a periodontal probe at 18 measurement points in 31 subjects	Overall = n/a
				P1 = 2.4 (± 0.6) to 3.9 (± 0.6)
				M2 = 2.6 (± 0.8) to 3.5 (± 1.2)
Wara-aswapati et al. [38]	*Thickness of palatal masticatory mucosa associated with age*	2001	Direct bone sounding with a periodontal probe in 62 subjects	Overall = 3.0 (± 0.3)
				P1 = 3.1 (± 0.5)
				M2 = 3.8 (± 1.2)
Barriviera et al. [23]	*A new method to assess and measure palatal masticatory mucosa by cone-beam computerised tomography*	2009	Punctual linear measurements of palatal soft tissue in 2D coronal CBCTs from 31 subjects	Overall = n/a
				P1 = 3.11
				M2 = 3.15
Yilmaz et al. [24]	*Cone-beam computed tomography evaluation of the soft tissue thickness and greater palatine foramen location in the palate*	2015	CBCT images of 345 subjects were measured in coronal slices	Overall = n/a
				P1 = 3.0 (± 0.75)
				M2 = 3.7 (± 0.48)
Hormdee et al. [25]	*Palatal soft tissue thickness on maxillary posterior teeth and its relation to palatal vault angle measured by cone beam computed tomography*	2020	Punctual, linear analysis of soft tissue thickness on the basis of CBCT data acquired in 2D coronal sections in 56 subjects	Overall = 3.71 (± 0.65)
				P1 = 3.9 (± 0.73)
				M2 = 3.86 (± 1.46)
Ogawa. et al. [26]	*Accuracy of cone beam computed tomography in evaluation of palatal mucosa thickness*	2020	Evaluations of palatal mucosa thickness in CBCT plus digital impressions in 174 subjects (after creating a conversion formula, based on direct K file measurements at 15 sites in 10 subjects)	Overall = n/a
				P1 = 3.20 (± 0.78) to 4.73 (± 0.76)
				M2 = 3.40 (± 1.32) to 5.74 (± 1.50)
Song et al. [27]	*Thickness of posterior palatal masticatory mucosa: the use of computerised tomography*	2008	Linear punctual measurement of palatal masticatory mucosa thickness at 24 measurement points on CT images from 100 subjects	Overall = 3.83 (± 0.58)
				P1 = 3.66 (± 0.66)
				M2 = 3.39 (± 1.00)
Uchida et al. [39]	*Measurement *in vivo* of Masticatory mucosal thickness with 20 MHz B-mode ultrasonic diagnostic equipment*	1989	Punctual measurements of palatal masticatory mucosa thickness with a 20-MHz B-Mode ultrasonic device in 100 edentulous patients	Overall = n/a
				P1 = 4.52 (± 0.9) to 4.46 (± 0.82)
				M2 = 5.62 (± 1.0) to 5.53 (± 0.91)
Müller et al. [18]	*Thickness of masticatory mucosa*	2000	Mucosa thickness measured in 40 subjects with a 5-MHz ultrasonic device	Overall = n/a
				P1 = 2.4 (± 0.45) to 2.89 (± 0.64)
				M2 = 2.44 (± 0.89) to 2.76 (± 1.15)
Schulze et al. [28]	*B-mode versus A-mode ultrasonographic measurements of mucosal thickness *in vivo	2002	Assessment of palatal masticatory mucosa with A-mode and B-mode, 10-MHz ultrasonographic devices in 50 subjects	Overall = 1.9 mm to 5.9 mm (95% CI, 2.9 to 3.3; median 3.1)
				P1 = n/a
				M2 = n/a
Heil et al. [41]	*Determination of the palatal masticatory mucosa thickness by dental MRI: a prospective study analysing age and gender effects*	2018	Assessment of palatal mucosa thickness on dental MRIs, at 3 Tesla with contrast-enhanced, high-resolution 3D-sequences; 40 measurement points in 40 subjects	Overall = 3.29 (± 0.45)
				P1 = 2.1 (± 0.1) to 4.5 (± 1.0)
				M2 = 2.1 (± 0.1) to 4.9 (± 0.8)

The results of our linear measurements (MeanDist, Table 2) were consistent with the trend of palatal soft tissue thicknesses shown in Table 3. Our linear measurements of 1.9–4.0 mm were comparable to the mean values of direct measurements (2.0–3.7 mm) reported in a study that performed direct probing in 62 subjects [38]. Our proposed 3D virtual analysis (Fig. 4) was consistent with reports in the literature that showed that the soft tissue thicknesses tended to increase from the anterior to the posterior palate [27, 38]. It should be noted that our evaluations included the masticatory mucosa of both the lateral palate and the posterior palate. Consistent with the findings of Studer et al. [15], our results showed that the keratinised mucosa was significantly thicker at the tuberosity and posterior palate (ROI 5, Table 2; Fig. 3) than in the rest of the hard palate. Furthermore, our results showed that the standard deviations of both the MeanDist and the Vol increased from anterior to posterior. This result suggested that tissue thicknesses showed greater anatomical variation near the tuberosity and the entry of the vascular-nerve bundle, compared to other regions. This observation was reported in most of the studies shown in Table 3 (compare the standard values for first premolar [P1] region to those of the second molar [M2] region). Wara-aswapati et al. [38] found that the overall thickness of the palatal masticatory mucosa increased from the gingival margin to the lateral palate, and then, decreased to the midline. That finding explained the variation in our results within a single segment, because the soft tissue volume at the midpalate is lower than the volume at the lateral palate, as shown in the colour-coded distance maps (Fig. 3), where the red colour indicates the largest volume and the green colour indicates no volume (i.e., contacting surfaces).

A spatial concept of each patient’s individual anatomy, as displayed in Fig. 3, prior to surgery has advantages. Intraoperative arterial bleeding and postoperative haemorrhage are risks that may arise when autologous grafts are extracted from the lateral palate, due to the proximity of the greater palatine artery. The digital analysis presented here showed that the highest volume was found in the most dorsal part of the hard palate on both sides of the maxilla (Fig. 3). This site coincides with the location of the vascular-nerve bundle that exits the greater palatine foramen. That bundle is typically described as distal to the third molar or between third and second molars, about 10–14 mm from the gingival margin [42]. These anatomical features were also found in the measurements acquired in this study; however, the findings must be interpreted with caution. For example, ROI 5 showed particularly high volume (Table 2), because the measurement included both the actual soft tissue volume and the volume of the palatal vascular-nerve bundle.

There are indications that may require a particularly thick soft tissue graft, such as implant-related or pre-prosthetic scenarios or to compensate tissue volume loss after a tooth extraction. In these cases, a presurgical soft tissue volume analysis might be beneficial. However, it is not sufficient to analyse solely the quantity of soft tissue; previous research has shown that good tissue quality (e.g., tissue rich in lamina propria) is crucial for surgical success. The layers of palatal soft tissue include the epithelium (approximately 0.30 – 0.44 mm thick), the connective tissue with the lamina propria (0.8 – 1.5 mm thick), and the submucosal tissue with its fatty component [43, 44]. It should be highlighted that the present study focused on the quantitative analysis of palatal soft tissue with 3D imaging to provide information about the anatomical conditions of the entire palate, but it did not investigate tissue quality.

Table 3 shows that examining radiological data in sectional views is a popular tool for soft tissue analysis. Some studies measured the palatal masticatory mucosa thickness in 2D sectional views of CBCTs. Ogawa et al. showed that punctual linear measurements on CBCTs were 0.34 (± 0.04) mm smaller than direct measurements with a K-file [26]. Considering the low contrast resolution, in general, soft tissue analyses are limited with CBCTs [45]; therefore, soft tissue measurements are only suitable to a limited extent in CBCTs. Consequently, we integrated a surface scan into our method of measuring soft tissue, by superimposing an intraoral surface model and a CBCT model.

As stated, there is a need for an easy, indirect, readily available method for measuring the soft tissue of the palate. The tissue volume measurement procedure performed in this study was indirect and non-invasive. Although a CBCT was used, it was not obtained for study purposes, but for navigated implant surgery or another medically justified indication. CBCT scans have become an indispensable part of everyday clinical practice in the field of oral and craniofacial surgery, but of course, the cost–benefit for the patient must be weighed, and radiological exposure should only be applied when justified [46].

Compared to CBCT, magnetic resonance imaging (MRI) might be superior for visualising intraoral hard and soft tissues while providing reliable data [41]. MRI has not been clinically established for dental purposes, it is expensive and requires a long examination time. Therefore, MRI is not currently an option for replacing the radiation emitting imaging of CBCT and MSCT. Mucosa measurements performed with MRI provide results comparable to those obtained with direct bone sounding [47], and MRI is the only absolutely non-invasive method, with no radiation risk.

The individual steps applied in this study were based on previous research that examined the accuracy of each step. The precision of the high-performance industrial optical scanner (Atos SO II, GOM GmbH) for digitising the impressions was 3 µm [34], and the accuracy of the converted 3D CBCT models was 400 (± 229) µm for 0.3 voxels [48]. The merging workflow was performed in a precise, two stage approach (manually and with a best-fit-algorithm), where images were registered by the tooth surfaces. Accurate imaging and precise superimposition are the foundation for the proposed workflow and reliable results. A recent study found that the matching accuracy was 300 µm with manual alignments of data (CBCT scan + surface scan) from patients without metallic restorations in the region of interest [32]. Data merging via the tooth surfaces was particularly accurate for patients without metallic restorations; therefore, the presence of a metallic restoration was selected as an exclusion criterion for participating in this study. A valid, reliable alternative to taking impressions of patients with high-precision silicone in this study would have been a direct intraoral scan as intraoral scans can correctly display both the teeth and the soft tissues [49, 50].

Merging 3D data provides information about anatomy, aesthetics, and function, before therapy has begun. In the field of digital dentistry, innovations arise at a fast pace, and they constantly lead to the development of therapy concepts and materials for oral rehabilitation based on 3D datasets. Recent innovations have included a fully digital workflow for dental prostheses [51, 52] and the emergence of highly functional materials, like oxide ceramics [53], fibre-reinforced composites [54] and glass/carbon fibres [55].

None of the studies listed in Table 3 measured soft tissue volume (in mm^3^). Therefore, no direct comparisons could be made with previous findings. Nevertheless, the results of this study should be interpreted with caution, because the sample size limited the reliability of the obtained measurements. It should be noted that this study was the first to test the possibility of 3D volumetric measurements of palatal soft tissue.

However, a 3D examination of soft tissue *changes* has been established and is currently used in clinical research [56]: That investigation technique involves superimposing pre- and postoperative intraoral surface scans or model scans, and then comparing them to determine the average tissue thickness and volume relative to baseline (i.e., preoperative values). For example, a recent study investigated the changes in palatal soft tissue, in terms of wound healing, after graft harvesting [57]. However, that technique solely compared the gingival surface at different time points, and CBCT scans were not integrated; therefore, only alterations in soft tissue relative to baseline were analysed. In contrast, our study showed that the proposed method could be used to determine, volumetrically, the *absolute* measurements of the soft tissue in the palate.

## Conclusions

Surgical planning software applies the principle of merging STL models via relevant structures with automatically generated HU-thresholds; however, to date, soft tissue assessments have not been performed.With the technique described in this study, the anatomical soft tissue features of the palate of each individual patient were displayed in three dimensions for preoperative planning. The superimposed bone and soft tissue data were used to fully exploit the diagnostic possibilities offered by 3D datasets. We showed that the measured soft tissue volume increased from the anterior (351 mm^3^) to the posterior palate (1863 mm^3^), with the greatest variance in the posterior palate. With this digital, indirect measurement method, based on merging 3D CBCT data and intraoral surface models, a projection of the palatal soft tissue volume was performed reliably for each patient individually. In future, studies are needed with a larger number of participants to determine the most appropriate harvesting site for maxillary grafts and to establish the presented digital method in clinical practice.

## Data Availability

All data generated or analysed during this study are included in this published article.

## Abbreviations

2D: Two-dimensional
3D: Three-dimensional
CBCT: Cone-Beam Computed Tomography
MSCT: Multi-Slice Computed Tomography
CAD/CAM: Computer-aided design/ Computer-aided manufacturing
STL: Standard Tessellation Language
DICOM: Digital Imaging and Communications in Medicine
HU: Hounsfield Units
ROI: Region of interest
MRI: Magnetic resonance imaging
